# Diverse human and bat-like rotavirus G3 strains circulating in suburban Bangkok

**DOI:** 10.1371/journal.pone.0268465

**Published:** 2022-05-24

**Authors:** Fajar Budi Lestari, Sompong Vongpunsawad, Yong Poovorawan

**Affiliations:** 1 Interdisciplinary Program of Biomedical Sciences, Faculty of Graduate School, Chulalongkorn University, Bangkok, Thailand; 2 Department of Bioresources Technology and Veterinary, Vocational College, Universitas Gadjah Mada, Yogyakarta, Indonesia; 3 Center of Excellence in Clinical Virology, Faculty of Medicine, Chulalongkorn University, Bangkok, Thailand; Consejo Superior de Investigaciones Cientificas, SPAIN

## Abstract

Although rotavirus vaccines are available in many parts of the world and are effective in reducing the overall incidence of rotavirus infection, it remains a major cause of diarrhea in less-developed countries. Among various rotavirus group A (RVA) strains, the increasingly common genotype G3 (defined by the VP7 gene) has been identified in both humans and animals. Our previous epidemiological surveillance in Bangkok found several unusual non-vaccine-like G3 strains in patients with diarrhea. In this study, we sequenced and characterized the genomes of seven of these G3 strains, which formed combinations with genotypes P[4], P[6], P[9], and P[10] (defined by the VP4 gene). Interestingly, we identified a bat-like RVA strain with the genome constellation G3-P[10]-I3-R3-C3-M3-A9-N3-T3-E3-H6, which has not been previously reported in the literature. The amino acid residues deduced from the nucleotide sequences of our G3 strains differed at the antigenic epitopes to those of the VP7 capsid protein of the G3 strain in RotaTeq vaccine. Although it is not unusual for the segmented genomes of RVA to reassort and give rise to emerging novel strains, the atypical G3 strains identified in this study suggest possible animal-to-human RVA zoonotic spillover even in urban areas.

## Introduction

Rotavirus is a major cause of viral diarrhea in very young children. Infection accounts for approximately 120,000 deaths annually in children younger than 5 years of age, the majority of whom live in developing countries [1]. There are designated 9 distinct rotavirus species (A-D and F-J) and 2 tentatively proposed species K and L, of which rotavirus group A (RVA) most often cause infection in humans [2, 3].

RVA belongs to the family *Reoviridae* and possesses segmented double-stranded RNA genome [4]. The virion is non-enveloped and has a triple-layered capsid. The 11 genomic RNA segments encode viral structural (VP1 to VP4, VP6, and VP7) and non-structural (NSP1 to NSP5) proteins [5]. The Rotavirus Classification Working Group (RCWG) designates the 11 RV genome segments encoding VP7-VP4-VP6-VP1-VP2-VP3-NSP1-NSP2-NSP3-NSP4-NSP5 into the corresponding genotype constellation Gx-P[x]-Ix-Rx-Cx-Mx-Ax-Nx-Tx-Ex-Hx, respectively [6]. To date, there are 41 G, 57 P, 31 I, 27 R, 23 C, 23 M, 38 A, 27 N, 27 T, 31 E, and 27 H genotypes among the RVA identified in human and animal species worldwide [7]. For most epidemiological surveillance of rotavirus, RVA genotype is often described by identifying the sequence of the VP7 glycoprotein (G) and VP4 protease-sensitive (P) protein [8].

RVA segmented genomes are amenable to reassortment, often resulting in strains with new genetic and antigenic properties [9, 10]. Due to the diversity of species RVA can infect, spillover infection of animal RVA into humans are not uncommon. Detection of such infection often requires extensive sequencing and determination of the viral genome constellation [8, 11–14], which can help determine the shared origin and evolutionary relationships among different RVA strains [15].

RVA of genotype G3 is one of the frequently detected strains worldwide [16–18]. The broad host range for G3 including feline, canine, equine, porcine, lapine, bovine, rats, rhesus, and bats has resulted in the occasional detection of animal-like RVA infection in patients with diarrhea [14, 19–24]. Although G3 is often found in combination with P[8], P[4], and P[6], pairings with P[9] and P[10] are less frequently reported [17, 21, 25–29]. Previously, we conducted surveillances of RVA infection in Thailand from 2015 to 2019 and found that G3 strains were most predominantly detected in adults and children with viral diarrhea [30–32]. Of interests are several atypical G3 strains not associated with the rotavirus vaccine strain, which could have arisen from zoonotic transmission. In this study, we characterized seven uncommon G3 strains from children and adults with RVA infection living in Bangkok.

## Materials and methods

### RVA genotyping

Among the RVA strains identified in patients with diarrhea during 2016–2018 [31, 32], seven unusual G3 strains initially determined based on the partial nucleotide sequence information were examined (strains B5383, B5356, B4401, B5368, B2682, B4684, and B5662). Archived viral RNA samples were subjected to conventional reverse-transcription polymerase chain reaction (RT-PCR) as previously described [33], including the use of additional primers to amplify the VP4 gene of P[10] (S1 Table). The PCR conditions were 40 cycles of denaturation at 94°C for 30 sec., annealing at 56°C for 45 sec., and extension at 72°C for 2 min. Amplicons were treated with ExoSAP (GE Healthcare, USA) prior to Sanger sequencing. Nucleotide sequences were analyzed using BioEdit [34] and subjected to BLAST search to yield percent nucleotide sequence identities (www.ncbi.nlm.nih.gov). Genotyping of all 11 gene segments was performed using the Rotavirus A Genotype Determination available through the Virus Pathogen Resource (ViPR) website https://www.viprbrc.org/brc/rvaGenotyper.spg) [35]. In addition to the previously deposited nucleotide sequences in the GenBank database, additional sequences were submitted under the accession numbers MW720858-MW720877, OK243970-OK243979, and OK244000-OK244038.

### Nucleotide sequence analysis

Gene segments were aligned with the RVA reference sequences by using ClustalW. Maximum-likelihood phylogenetic trees were constructed with the best substitution models determined based on the corrected Bayesian information criterion value implemented in MEGA7 [36, 37]. The models used in this study were General Time Reversible (GTR) + gamma distributed (G) + invariable sites (I) (for VP1, VP2, VP3, and NSP1), Tamura 3-parameter (T92) + G + I (for VP4, VP6, VP7, NSP2, NSP3, and NSP5) and T92 + G (for NSP4). Tree robustness was determined by bootstrapping of 1,000 replicates with values >70% considered significant.

### Amino acid residue analysis

Deduced amino acid residues from the nucleotide-sequenced strains were aligned with those of the RVA vaccine G3 in RotaTeq. Changes were mapped onto the published structure of trimeric VP7 (Protein Data Bank number 3FMG) using PyMOL software.

## Results

Seven G3 strains (B5383, B5356, B4401, B5368, B2682, B4684, and B5662) in various combinations with P[4], P[6], P[9], and P[10] were from patients 2 to 49 years of age of both genders, only two of whom are children (<4 years old) (Table 1). These samples were from infection detected from December to May of each year, which coincide with the typical annual RVA season in Thailand.

**Table 1:** Description of the RVA samples in this study.

Strain designation	Collection date	Gender	Age
RVA/Human-wt/THA/B5383/2018/G3P[4]	06/03/18	F	34 yr 11 mo
RVA/Human-wt/THA/B5356/2018/G3P[4]	02/03/18	F	29 yr 6 mo
RVA/Human-wt/THA/B4401/2017/G3P[6]	23/12/17	M	3 yr 11 mo
RVA/Human-wt/THA/B5368/2018/G3P[6]	05/03/18	F	49 yr 4 mo
RVA/Human-wt/THA/B2682/2016/G3P[9]	24/03/16	M	2 yr
RVA/Human-wt/THA/B4684/2018/G3P[10]	25/01/18	M	34 yr 7 mo
RVA/Human-wt/THA/B5662/2018/G3P[10]	03/05/18	F	44 yr 10 mo

F, female; M, male; yr, years; mo, months.

### RVA genome constellation

To further characterize these atypical G3 strains, we determined the near-complete nucleotide sequences of all 11 gene segments. The genome constellations of the two G3P[4] (B5383 and B5356) and two G3P[6] (B4401 and B5368) mostly resembled the prototypic DS-1 (I2-R2-C2-M2-A2-N2-T2-E2-H2 (Table 2). Our G3P[6] strains, however, had identical genome constellation as RVA/Human-wt/IDN/STM182/2016/G3P[6] identified in Indonesia in 2016. Meanwhile, the G3P[9] mirrored the prototypic AU-1 in all gene segments except NSP5. Although 8 gene segments in the two G3P[10] (B4684 and B5662) shared similarities with AU-1, neither strains were identical to any established reference strains. When compared to global strains, however, they were genotypically identical in all gene segments except VP6 to the RVA/Bat-tc/CHN/MYAS33/2013/G3P[10] and a RVA/Human-wt/THA/MS2015-1-0001/2015/G3P[10], of which the latter was identified in 2015 from a patient who lived in northern Thailand.

**Table 2:** Genome constellations of RVA G3 characterized in this study (in italics) compared to several RCWG reference (in bold) and global strains.

Strain Name	Genotypes										
	VP7	VP4	VP6	VP1	VP2	VP3	NSP1	NSP2	NSP3	NSP4	NSP5
**RVA/Human-tc/USA/DS-1/1976/G2P[4]**	**G2**	**P[4]**	**I2**	**R2**	**C2**	**M2**	**A2**	**N2**	**T2**	**E2**	**H2**
*RVA/Human-wt/THA/B5383/2018/G3P[4]*	G3	P[4]	I2	R2	C2	M2	A2	N2	T2	E2	H2
*RVA/Human-wt/THA/B5356/2018/G3P[4]*	G3	P[4]	I2	R2	C2	M2	A2	N2	T2	E2	H2
*RVA/Human-wt/THA/B4401/2017/G3P[6]*	G3	P[6]	I2	R2	C2	M2	A2	N2	T2	E2	H2
*RVA/Human-wt/THA/B5368/2018/G3P[6]*	G3	P[6]	I2	R2	C2	M2	A2	N2	T2	E2	H2
RVA/Human-wt/IDN/STM182/2016/G3P[6]	G3	P[6]	I2	R2	C2	M2	A2	N2	T2	E2	H2
RVA/Human-wt/RUS/Novosibirsk/NS17-A1301/2017/G3P[6]	G3	P[6]	I2	Rx	Cx	Mx	Ax	N2	Tx	E2	H2
**RVA/Human-wt/JPN/AU-1/1982/G3P[9]**	**G3**	**P[9]**	**I3**	**R3**	**C3**	**M3**	**A3**	**N3**	**T3**	**E3**	**H3**
*RVA/Human-wt/THA/B2682/2016/G3P[9]*	G3	P[9]	I3	R3	C3	M3	A3	N3	T3	E3	H6
*RVA/Human-wt/THA/B4684/2018/G3P[10]*	G3	P[10]	I3	R3	C3	M3	A9	N3	T3	E3	H6
*RVA/Human-wt/THA/B5662/2018/G3P[10]*	G3	P[10]	I3	R3	C3	M3	A9	N3	T3	E3	H6
RVA/Bat-tc/CHN/MYAS33/2013/G3P[10]	G3	P[10]	I8	R3	C3	M3	A9	N3	T3	E3	H6
RVA/Human-wt/THA/MS2015-1-0001/2015/G3P[10]	G3	P[10]	I8	R3	C3	M3	A9	N3	T3	E3	H6
RVA/Human-wt/CHN/M2-102/2014/G3P[3]	G3	P[3]	I3	R3	C3	M3	A9	N3	T3	E3	H6
**RVA/Rhesus-tc/USA/TUCH/2002/G3P[24]**	**G3**	**P[24]**	**I9**	**R3**	**C3**	**M3**	**A9**	**N1**	**T3**	**E3**	**H6**

### Analyses using phylogenetic trees and pairwise comparisons

**Fig 1:**
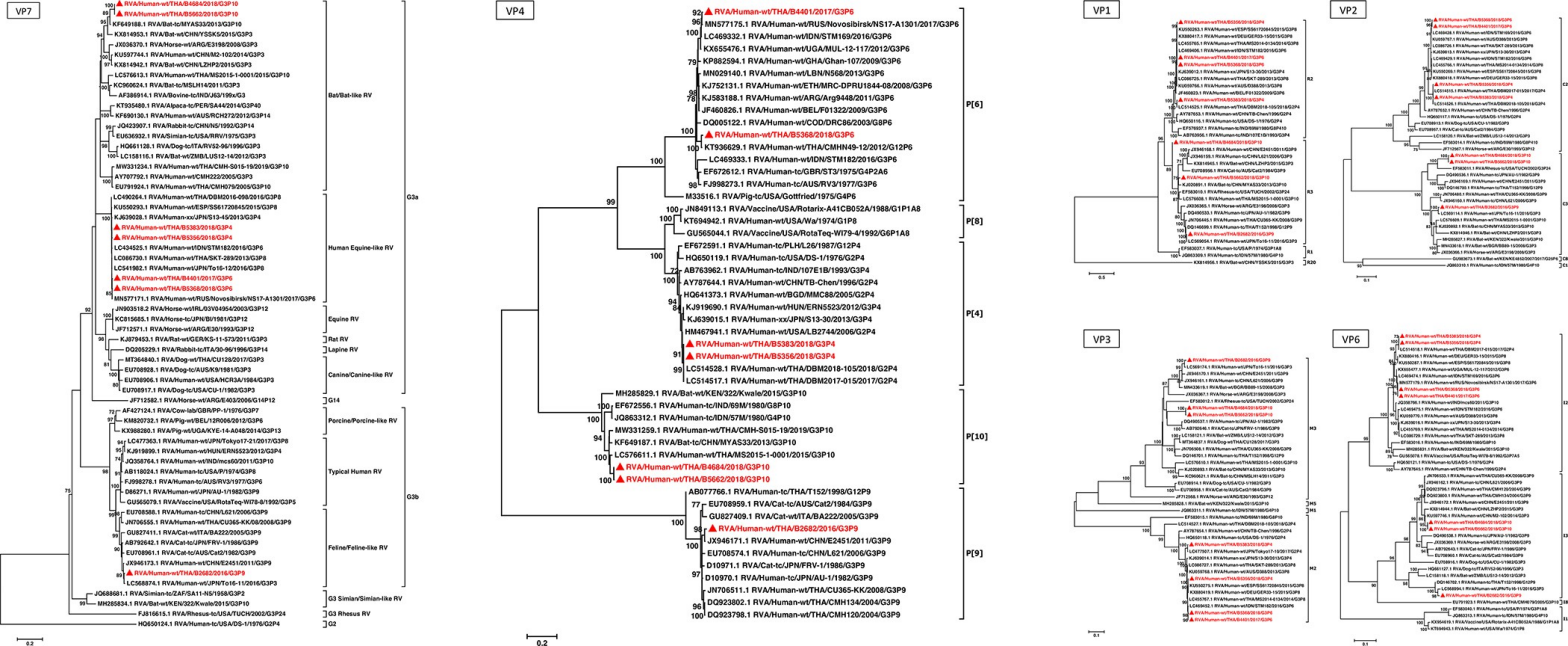
Phylogenetic analysis of the nucleotide sequences of G3 RVA structural protein genes. Thai strains (noted with triangles and in red) were compared to the reference and previously reported RVA strains. Nucleotide sequence lengths used in the maximum-likelihood phylogenetic analysis for VP1, VP2, VP3, VP4, VP6, and VP7 genes were 3204, 2528, 2507, 759, 1157, and 801 base pairs, respectively. Bootstrap values >70% are indicated at the tree nodes. Scale bars represent substitutions per nucleotide.

**Fig 2:**
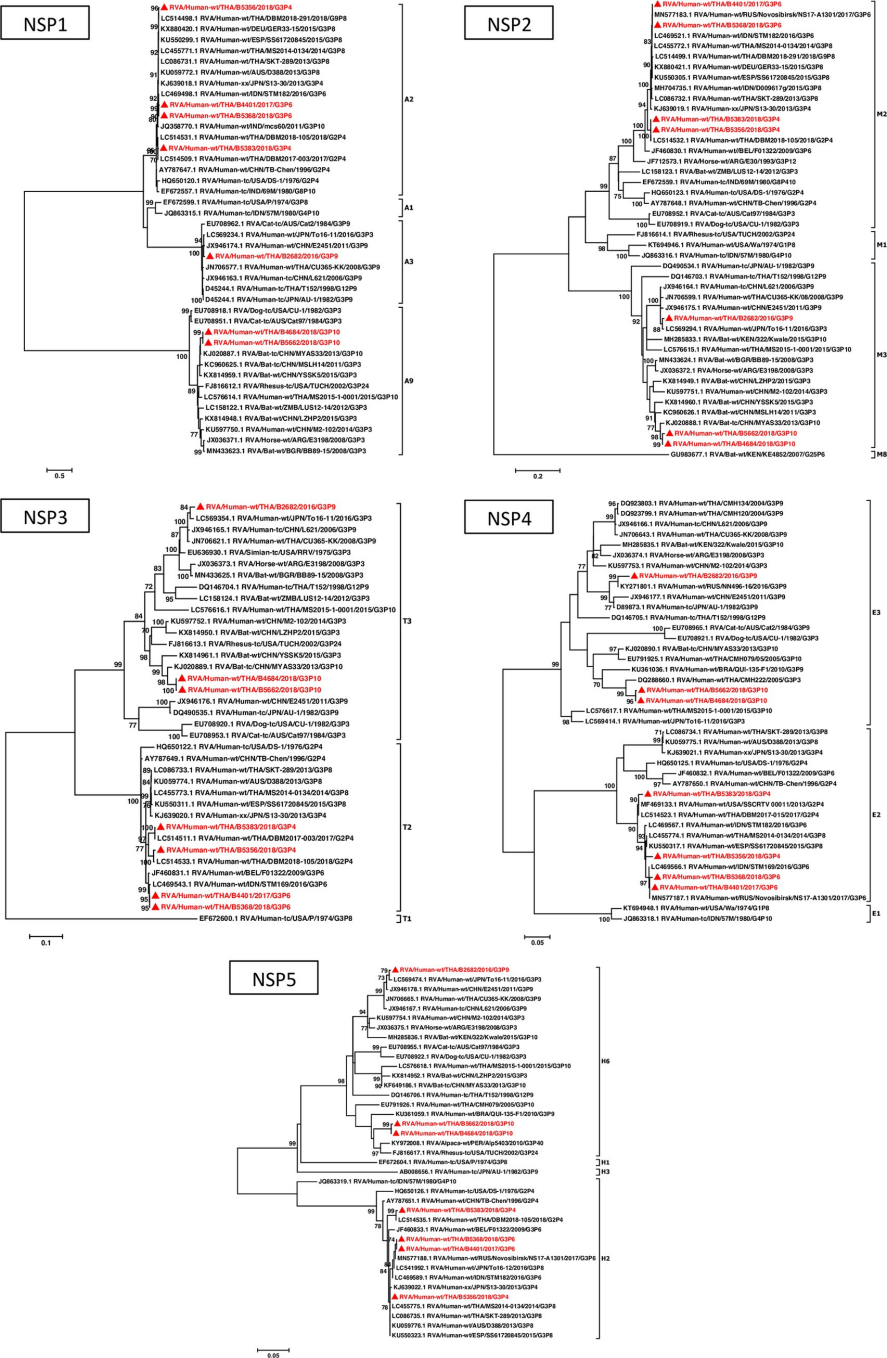
Phylogenetic analysis of the nucleotide sequences of G3 RVA non-structural protein genes. Thai strains (noted with triangles and in red) were compared to the reference and previously reported RVA strains. Nucleotide sequence lengths used in the maximum-likelihood phylogenetic analysis for NSP1, NSP2, NSP3, NSP4, and NSP5 genes were 1302, 953, 904, 618, and 520 base pairs, respectively. Bootstrap values >70% are indicated at the tree nodes. Scale bars represent substitutions per nucleotide.

To examine the extent of how our G3 strains were similar to the reference and global RVA strains, we performed phylogenetic analyses for all of their 11 gene segments (Figs 1 and 2). The VP7 gene of our G3P[4] and G3P[6] strains clustered with human equine-like RVA, while the G3P[9] clustered with the feline-like RVA. Our two G3P[10] strains grouped with the bat-like MYAS33 strain. Meanwhile, our VP4 gene sequences were similar to various global strains. In particular, one of the two G3P[6] strains appeared closest to RVA/Human-wt/RUS/Novosibirsk/NS17-A1301/2017/G3P[6], while both of our G3P[10] strains were very similar to the MS2015-1-0001 and MYAS33 strains.

Pairwise comparison of the nucleotide sequences of our G3P[4] and G3P[6] strains were ≥99% identical to the gene segments of global RVA strains (S2 Table). Our G3P[9] also shared >97% sequence identities with other previously described strains. In contrast, nucleotide identities for some of the G3P[10] gene segments were lower. While the VP6 gene of our G3P[10] strains were >98% identical to the M2-102 strain identified in China, the VP3 genes were only 88% identical to the closest known relative RVA/Bat-wt/ZMB/LUS12-14/2012/G3P[3]. Moreover, the VP1 gene of G3P[10] strain B4684 (89%) was lower than B5662 (93%) when compared to the most similar MYAS33 strain.

### Deduced amino acid residues of G3 strains mapped onto the VP7 trimer

**Fig 3:**
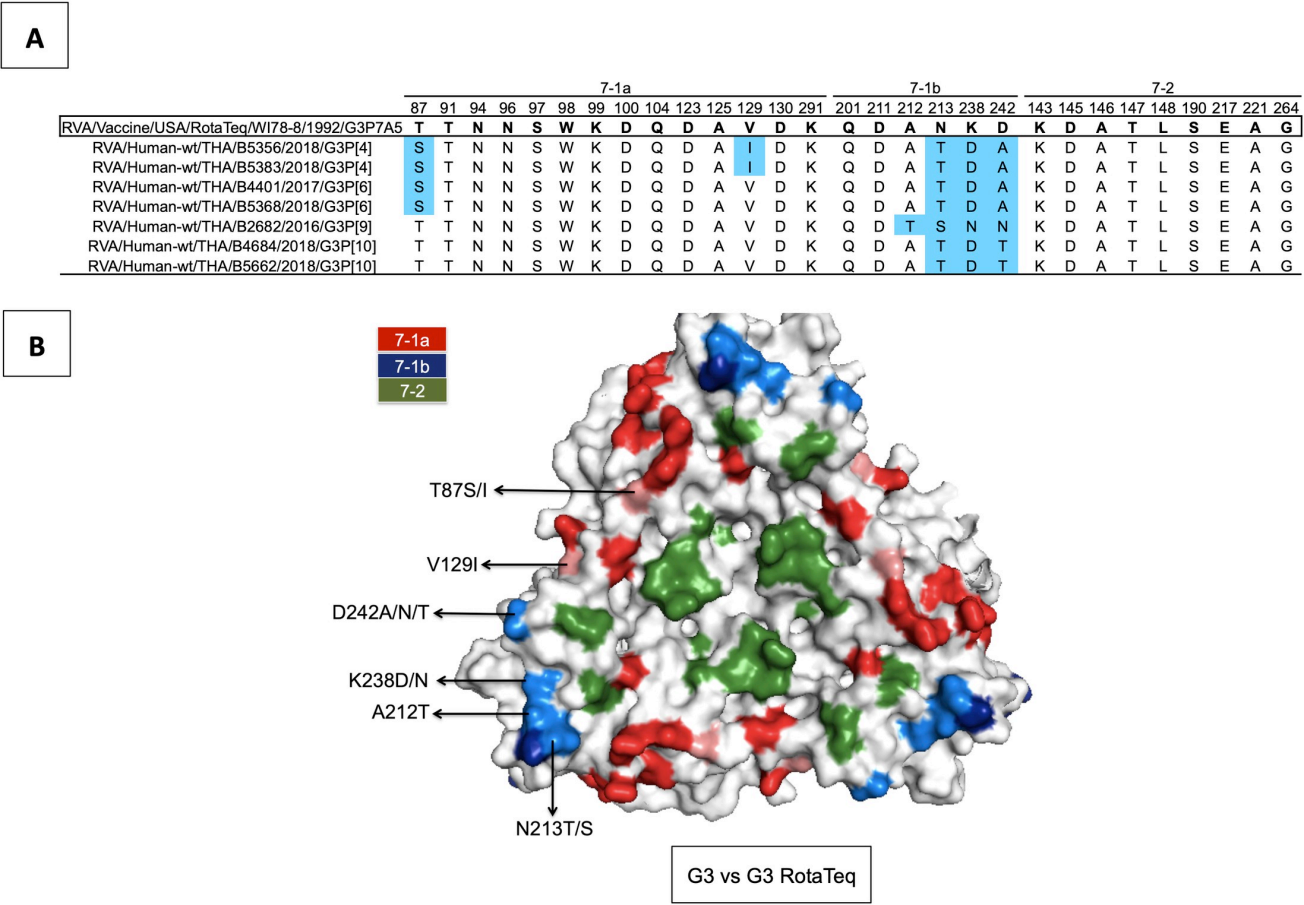
Deduced VP7 amino acid residues of G3 strains mapped to the surface of the vaccine G3 VP7 trimer. (A) Alignment of the VP7 residues comprising the antigenic epitopes (7-1a, 71b, and 7–2). Residues different from RotaTeq are shaded light blue. (B) Surface representation of the VP7 trimer (PDB 3FMG). Antigenic epitopes are colored red (7-1a), blue (7-1b), and green (7–2). Surface-exposed residue differences between our G3 strains and G3 RotaTeq are lighter in color.

VP7 has several defined antigenic epitopes (designated 7-1a, 7-1b, and 7–2). To determine amino acid changes between our G3 and the RotaTeq G3 vaccine strains and where these changes are located, we performed a sequence alignment. Although most antigenic residues were identical, notable differences were observed on residues 87 and 129 in epitope 7-1a, and residues 212, 213, 238, and 242 in epitope 7-1b (Fig 3). Specifically, G3P[4] possessed T87S and V129I (on epitope 7-1a) and N213T, K238D, and D242A (on epitope 7-1b). G3P[6] possessed all of the above changes except residue 129, which was identical to the vaccine strain. Moreover, G3P[9] differed from the vaccine strain only on epitope 7-1b (A212T, N213S, K238N, and D242N). Finally, G3P[10] was characterized by N213T, K238D, and D242T on epitope 7-1b.

## Discussion

RVA infection is traditionally associated with very young children, but occasional infection among adults do occur [30, 31]. In this study, we characterized the near-complete genome of seven atypical G3 strains, many of which were identified in adults who were unlikely to have been rotavirus-vaccinated. G3 strains represent diverse genome constellations with currently up to 20 RCWG-established genotypes [6]. The uncommon RVA strains from five infected adults versus two children may be attributed to adults’ increased risk of infection with increasing age. Additional factors may also include occupational exposure, travel, or other life activities. Although patients from whom samples were derived live in and around Bangkok and likely did not have frequent contacts with wildlife, it is interesting to note that many of the gene segments closely resembled RVA of animal origins.

The G3P[4] and G3P[6] strains possessed equine-like G3, which was first detected in Japan in 2013 [20] and has since become predominant worldwide [14, 38–43]. They possess similar genome constellation as the prototypic DS-1 except for the VP7 gene. From our analysis, they also exhibit high degree of identity (99–100%) with equine-like G3 from Russia (Novosibirsk/NS17-A1301) and Indonesia (STM182) [44].

Early detection of G3P[9] as represented by the prototypic AU-1 was from a patient in Japan in 1982 [45]. Human G3P[9] strains including our B2682 share common origins with feline RVA [28, 46, 47] and are infrequently detected in human [18, 48–52]. However, G3P[9] occasionally surfaced in Thailand (CMH120/04, CMH134/04, CU365) [25, 28]. The B2682 possessed identical genome constellation G3-P[9]-I3-R3-C3-M3-A3-N3-T3-E3-H6 as another Thai strain CU365 previously reported by our group [28] and strains L621 and E2451 from China [53]. It was observed that all G3P[9] strains regardless of host species possess an A3 NSP1 gene. This unique combination of P[9] and A3 was hypothesized to provide a replication advantage in various hosts [54].

Finding of G3P[10] in diarrheic adults living in Bangkok was unexpected. This genotype first appeared in 2005 during an epidemiological surveillance and was identified from a 2-year-old child hospitalized for severe diarrhea in northern Thailand [26]. Designated CMH079, this strain was not completely characterized at the time and lacked genotype constellation information. Subsequent reports of G3P[10] were from a diarrheic 14-month-old child in eastern India (mcs60 strain) in 2011, and again in Thailand from an 11-month-old infant (MS2015-1-0001 strain) in 2015 [21, 55]. The most recently reported G3P[10] infection in Thailand was in a 1 year-old (CMH-S015-19) in January 2019 [56].

In our study, clinical symptoms reported by both adult patients with G3P[10] (B4684 and B5662) included fever, watery diarrhea, abdominal pain, vomiting, and dehydration. Although B4684 and B5662 were identified four months apart, their VP1 nucleotide sequences were sufficiently different to the genetically closest MYAS33 strain from bat (at 89% vs. 93%, respectively) that they are unlikely to be epidemiologically linked. Interestingly, the genome constellation of our G3P[10] and several previously described global strains differed by a single gene segment, either VP4 or VP6. For instance, our G3P[10] resembled the bat LZHP2, BB89-15, and BR89-60 strains, human M2-102 strain, and equine E3198 strain in all gene segments except VP4. Alternatively, B4684 and B5662 may be associated with another human strain MS2015-1-0001 first reported in northern Thailand. If so, the VP6 I3 in their genome constellation G3–P[10]–I3–R3–C3–M3–A9–N3–T3–E3–H6 had replaced the I8 in MS2015-1-0001 within the span of three years. It is noteworthy that the latter has an identical genotype constellation as a bat RVA strain MYAS33 identified in 2013 in China [57], which suggests probable zoonotic origin.

RotaTeq is a live attenuated vaccine containing reassortant strains of genotypes G1, G2, G3, G4, and P[8] [58]. It was initially feared that the rotavirus vaccines may impose selective pressure on circulating RVA strains, possibly influencing their evolutionary rate and the transmissibility of new RVA strains [59]. Moreover, the global emergence of equine-like G3 DS-1-like strains raised questions of whether vaccinations induced selective pressure on zoonotic RVA strains [60]. However, the use of rotavirus vaccines in Thailand is currently not widespread and Thai adults are extremely unlikely to have been vaccinated. When we mapped the deduced amino acid residue changes of our G3 strains onto the trimeric VP7 protein structure of the vaccine G3 strain, we identified several differences on the antigenic epitopes. Many of the differences were P type-specific, such as V129I in 7-1a for G3P[4] or D242T in 7-1b for G3P[10]. Residues of our G3P[9] differed most from the vaccine, particularly on the 7-1b antigenic epitope. Interestingly, the K238N substitution, which is also present in various G3 strains from Argentina, Belgium, Pakistan, and Lebanon [16, 17, 61–63], can potentially introduce an N-linked glycosylation in the 7-1b epitope and reduce antibody neutralization [64].

This study has several limitations. We do not know whether there are unreported RVA infection of G3 genome constellation similar to ours elsewhere in Bangkok because RVA genome characterization is not typically done for viral diagnostics. Diarrheic adults do not always seek medical attention and only do so when symptoms are severe, therefore unreported cases may exist. We also do not know how our patients were infected as this information cannot be ascertained from the medical records at hand. It would be helpful to investigate whether any households of these patients had pets or other animals, which were also infected with RVA, to further examine animal-to-human transmission.

In conclusion, the G3 RVA strains identified in this study highlight unusual RVA genotype constellations even in urban settings, which would have otherwise eluded detection without thorough genome analysis. Continued surveillance and molecular characterization of novel RVA in both human and animals are important to understand possible zoonosis and future strain inclusions in the vaccine.

## Supporting information

S1 Table Oligonucleotide primers used to amplify the VP4 segment of P[10]. (DOCX)

S2 Table Nucleotide sequence identities between 7 G3 RVA strains and their closest related strains for all gene segments. (DOCX)
