# Supplementary material for: Diverse human and bat-like rotavirus G3 strains circulating in suburban Bangkok
Source: PLoS One. 2022 May 24;17(5):e0268465. doi: 10.1371/journal.pone.0268465 (PMC9129036; doi:10.1371/journal.pone.0268465)
Supplement: S1 Table — (DOCX) [file pone.0268465.s001.docx]

S1 Table. Oligonucleotide primers used to amplify the VP4 segment of P[10].

| Primer name | Sequence (5' to 3') | Position* |
| --- | --- | --- |
| VP4_P10_F2 | TGGCTTCGCTCATTTACAGAC | 2-22 |
| VP4_P10_R1153 | ACTGGCAATGCGAAACTGTA | 1134-1153 |
| VP4_P10_F987 | TGGTGGATCATTACCAACTGAC | 987-1008 |
| VP4_P10_R2260 | CTCGTAGCACTCTAGGATCAGA | 2239-2260 |

*Relative to the MYAS33 strain (GenBank accession number KF649187).
