# Supplementary material for: Diverse human and bat-like rotavirus G3 strains circulating in suburban Bangkok
Source: PLoS One. 2022 May 24;17(5):e0268465. doi: 10.1371/journal.pone.0268465 (PMC9129036; doi:10.1371/journal.pone.0268465)
Supplement: S2 Table — (DOCX) [file pone.0268465.s002.docx]

S2 Table. Nucleotide sequence identities between 7 G3 RVA strains and their closest related strains for all gene segments.

| **Strain Name** | **Gene** | **Strains that exhibit close nucleotide sequence identities** | **Identity (%)** |
| --- | --- | --- | --- |
| **RVA/Human-wt/THA/B5383/**  **2018/G3P[4]** | VP1 | RVA/Human-wt/THA/DBM2018-291/2018/G9P[8] | 99.82% |
|  | VP2 | RVA/Human-wt/THA/DBM2017-003/2017/G2P[4] | 99.96% |
|  | VP3 | RVA/Human-wt/JPN/Tokyo17-10/2017/G2P[4] | 99.42% |
|  | VP4 | RVA/Human-wt/THA/DBM2018-105/2018/G2P[4] | 99.86% |
|  | VP6 | RVA/Human-wt/THA/DBM2017-015/2017/G2P[4] | 99.92% |
|  | VP7 | RVA/Human-wt/IDN/D009617g/2015/G3P[8] | 99.90% |
|  | NSP1 | RVA/Human-wt/THA/DBM2017-003/2017/G2P[4] | 99.60% |
|  | NSP2 | RVA/Human-wt/THA/DBM2018-105/2018/G2P[4] | 99.90% |
|  | NSP3 | RVA/Human-wt/THA/DBM2017-003/2017/G2P[4] | 99.90% |
|  | NSP4 | RVA/Human-wt/USA/SSCRTV_00011/2013/G2P[4] | 99.86% |
|  | NSP5/6 | RVA/Human-wt/THA/DBM2017-003/2017/G2P[4] | 99.51% |
| **RVA/Human-wt/THA/B5356/**  **2018/G3P[4]** | VP1 | RVA/Human-wt/ESP/SS96217158/2015/G3P[8] | 99.51% |
|  | VP2 | RVA/Human-wt/THA/DBM2017-015/2017/G2P[4] | 99.96% |
|  | VP3 | RVA/Human-wt/ESP/SS61720845/2015/G3P[8] | 99.81% |
|  | VP4 | RVA/Human-wt/THA/DBM2018-105/2018/G2P[4] | 100.00% |
|  | VP6 | RVA/Human-wt/THA/DBM2017-015/2017/G2P[4] | 99.84% |
|  | VP7 | RVA/Human-wt/ESP/SS98242319/2015/G3P[8] | 99.71% |
|  | NSP1 | RVA/Human-wt/DEU/GER33-15/2015/G3P[8] | 99.66% |
|  | NSP2 | RVA/Human-wt/THA/DBM2018-105/2018/G2P[4 | 100.00% |
|  | NSP3 | RVA/Human-wt/THA/DBM2017-015/2017/G2P[4] | 99.70% |
|  | NSP4 | RVA/Human-wt/ESP/SS61720845/2015/G3P[8] | 99.33% |
|  | NSP5/6 | RVA/Human-wt/THA/DBM2018-291/2018/G9P[8] | 99.75% |
| **RVA/Human-wt/THA/B4401/**  **2017/G3P[6]** | VP1 | RVA/Human-wt/IDN/STM182/2016/G3P[6] | 99.85% |
|  | VP2 | RVA/Human-wt/IDN/STM169/2016/G3P[6] | 99.48% |
|  | VP3 | RVA/Human-wt/THA/MS2014-0134/2014/G3P[8] | 99.72% |
|  | VP4 | RVA/Human-wt/RUS/Novosibirsk/NS17-A1301/2017/G3P[6] | 99.53% |
|  | VP6 | RVA/Human-wt/RUS/Novosibirsk/NS17-A1301/2017/G3P[6] | 99.69% |
|  | VP7 | RVA/Human-wt/RUS/Novosibirsk/NS17-A1301/2017/G3P[6] | 99.90% |
|  | NSP1 | RVA/Human-wt/IDN/STM182/2016/G3P[6] | 98.99% |
|  | NSP2 | RVA/Human-wt/RUS/Novosibirsk/NS17-A1301/2017/G3P[6] | 99.90% |
|  | NSP3 | RVA/Human-wt/IDN/STM169/2016/G3P[6] | 99.61% |
|  | NSP4 | RVA/Human-wt/RUS/Novosibirsk/NS17-A1301/2017/G3P[6] | 100.00% |
|  | NSP5/6 | RVA/Human-wt/RUS/Novosibirsk/NS17-A1301/2017/G3P[6] | 99.86% |
| **RVA/Human-wt/THA/B5368/**  **2018/G3P[6]** | VP1 | RVA/Human-wt/IDN/STM182/2016/G3P[6] | 99.85% |
|  | VP2 | RVA/Human-wt/IDN/STM050/2015/G3P[8] | 99.47% |
|  | VP3 | RVA/Human-wt/THA/MS2014-0134/2014/G3P[8] | 99.69% |
|  | VP4 | RVA/Human-wt/THA/CMHN49-12/2012/G12P[6] | 99.05% |
|  | VP6 | RVA/Human-wt/RUS/Novosibirsk/NS17-A1301/2017/G3P[6] | 99.92% |
|  | VP7 | RVA/Human-wt/RUS/Novosibirsk/NS17-A1301/2017/G3P[6] | 99.89% |
|  | NSP1 | RVA/Human-wt/IDN/STM182/2016/G3P[6] | 99.46% |
|  | NSP2 | RVA/Human-wt/RUS/Novosibirsk/NS17-A1301/2017/G3P[6] | 99.70% |
|  | NSP3 | RVA/Human-wt/IDN/STM169/2016/G3P[6] | 99.22% |
|  | NSP4 | RVA/Human-wt/RUS/Novosibirsk/NS17-A1301/2017/G3P[6] | 99.16% |
|  | NSP5/6 | RVA/Human-wt/RUS/Novosibirsk/NS17-A1301/2017/G3P[6] | 99.61% |
| **RVA/Human-wt/THA/B2682/**  **2016/G3P[9]** | VP1 | RVA/Human-wt/JPN/To16-11/2016/G3P[3] | 98.95% |
|  | VP2 | RVA/Human-wt/JPN/To16-11/2016/G3P[3] | 98.80% |
|  | VP3 | RVA/Human-wt/JPN/To16-11/2016/G3P[3] | 98.45% |
|  | VP4 | RVA/Human-wt/CHN/E2451/2011/G3P[9] | 98.07% |
|  | VP6 | RVA/Human-tc/THA/T152/1998/G12P[9] | 98.24% |
|  | VP7 | RVA/Human-wt/JPN/To16-11/2016/G3P[3] | 98.32% |
|  | NSP1 | RVA/Human-wt/JPN/To16-11/2016/G3P[3] | 97.08% |
|  | NSP2 | RVA/Human-wt/JPN/To16-11/2016/G3P[3] | 98.88% |
|  | NSP3 | RVA/Human-wt/JPN/To16-11/2016/G3P[3] | 97.79% |
|  | NSP4 | RVA/Human-wt/RUS/NN496-16/2016/G3P[9] | 97.95% |
|  | NSP5/6 | RVA/Human-wt/JPN/To16-11/2016/G3P[3] | 99.14% |
| **RVA/Human-wt/THA/B4684/**  **2018/G3P[10]** | VP1 | RVA/Bat-tc/CHN/MYAS33/2013/G3P[10] | 89.41% |
|  | VP2 | RVA/Rhesus-tc/USA/TUCH/2002/G3P[24] | 91.32% |
|  | VP3 | RVA/Bat-wt/ZMB/LUS12-14/2012/G3P[3] | 88.06% |
|  | VP4 | RVA/Human-wt/THA/MS2015-1-0001/G3P[10] | 96.48% |
|  | VP6 | RVA/Human-wt/CHN/M2-102/2014/G3P[3] | 98.67% |
|  | VP7 | RVA/Bat-tc/CHN/MYAS33/2013/G3P[10] | 96.36% |
|  | NSP1 | RVA/Bat-tc/CHN/MYAS33/2013/G3P[10] | 94.09% |
|  | NSP2 | RVA/Bat-tc/CHN/MYAS33/2013/G3P[10] | 97.71% |
|  | NSP3 | RVA/Bat-tc/CHN/MYAS33/2013/G3P[10] | 95.53% |
|  | NSP4 | RVA/Human-wt/THA/CMH222/2005/G3P[3] | 96.36% |
|  | NSP5/6 | RVA/Alpaca-wt/PER/Alp5403/2010/G3P[40] | 95.74% |
| **RVA/Human-wt/THA/B5662/**  **2018/G3P[10]** | VP1 | RVA/Bat-tc/CHN/MYAS33/2013/G3P[10] | 93.04% |
|  | VP2 | RVA/Rhesus-tc/USA/TUCH/2002/G3P[24] | 91.45% |
|  | VP3 | RVA/Bat-wt/ZMB/LUS12-14/2012/G3P[3] | 88.17% |
|  | VP4 | RVA/Human-wt/THA/MS2015-1-0001/G3P[10] | 96.35% |
|  | VP6 | RVA/Human-wt/CHN/M2-102/2014/G3P[3] | 98.43% |
|  | VP7 | RVA/Bat-tc/CHN/MYAS33/2013/G3P[10] | 96.26% |
|  | NSP1 | RVA/Bat-tc/CHN/MYAS33/2013/G3P[10] | 94.43% |
|  | NSP2 | RVA/Bat-tc/CHN/MYAS33/2013/G3P[10] | 96.79% |
|  | NSP3 | RVA/Bat-tc/CHN/MYAS33/2013/G3P[10] | 95.43% |
|  | NSP4 | RVA/Human-wt/THA/CMH222/2005/G3P[3] | 96.50% |
|  | NSP5/6 | RVA/Alpaca-wt/PER/Alp5403/2010/G3P[40] | 95.33% |
